# Multi-Technique Assessment of Chelators-Loaded PVA-Borax Gel-like Systems Performance in Cleaning of Stone Contaminated with Copper Corrosion Products

**DOI:** 10.3390/gels10070455

**Published:** 2024-07-11

**Authors:** Laura Giuliani, Chiara Genova, Valeria Stagno, Luca Paoletti, Andrea Louise Matulac, Alessandro Ciccola, Melania Di Fazio, Silvia Capuani, Gabriele Favero

**Affiliations:** 1 Department of Earth Sciences, Sapienza University of Rome, P.le Aldo Moro 5, 00185 Rome, Italy; laura.giuliani@uniroma1.it (L.G.); melania.difazio@uniroma1.it (M.D.F.); 2 Department of Environmental Biology, Sapienza University of Rome, P.le Aldo Moro 5, 00185 Rome, Italy; chiara.genova@uniroma1.it (C.G.); alessandro.ciccola@uniroma1.it (A.C.); gabriele.favero@uniroma1.it (G.F.); 3 Department of Chemistry and Technologies of Drug, Sapienza University of Rome, P.le Aldo Moro 5, 00185 Rome, Italy; luca.paoletti@uniroma1.it; 4 Erasmus Mundus Joint Master’s Degrees Archmat, Sapienza University of Rome, P.le Aldo Moro 5, 00185 Rome, Italy; andreamatulac@gmail.com; 5 National Research Council Institute for Complex Systems (CNR-ISC) c/o Physics Department, Sapienza University of Rome, P.le Aldo Moro 5, 00185 Rome, Italy; silvia.capuani@isc.cnr.it

**Keywords:** portable NMR, SEM-EDS, FTIR, gel-like formulations, polyvinyl alcohol-borax systems, chelating agents, carbonate stones

## Abstract

Currently, one of the most important challenges for the conservation of stone artworks is the removal of metal corrosion products on their surfaces. Traditional cleaning methods, which typically involve the application of aqueous solutions containing chelating agents capable of complexing these metal ions, have shown some weaknesses. These weaknesses become apparent when such methods are applied to statues and other vertical surfaces or when aiming to limit the cleaning process to a specific area with controlled application times. Furthermore, the porosity of the stone surface plays a role concerning the cleaning efficiency. To address these issues, chelating agents can be incorporated into gel-like materials. This study is a proof of concept to evaluate the cleaning efficacy of various gel formulations composed of polyvinyl alcohol (PVA), borax (B), and agarose (AG), loaded with two chelators: ethylenediaminetetraacetic acid (EDTA) and potassium sodium tartrate (PST or Rochelle salt). Three types of carbonate stones (travertine, Lecce stone, and Carrara marble) characterized by different porosities were artificially stained with copper sulphates and treated with the different PVA-B-AG formulations. The effectiveness of the treatment was directly monitored on the stones using a multi-technique approach that included scanning electron microscopy with energy dispersive spectroscopy (SEM-EDS) and non-invasive portable nuclear magnetic resonance (NMR). Additionally, the rheological properties of the gels were investigated, and the Fourier transform infrared attenuated total reflection spectroscopy (FTIR ATR) was used to analyse the chemical structure of the gel before and after treatment, aiming to understand the changes induced by the cleaning process.

## 1. Introduction

Stone artworks constitute a valuable component of our cultural heritage, and it is imperative to safeguard them for future generations. Cleaning stone buildings, statues, or monuments demands meticulous care to prevent compromising their aesthetic and historical integrity. Safely cleaning historic stone without damaging its distinctive qualities entails several crucial steps, including identifying the stone, assessing its decay, and rigorously testing the cleaning method [1]. Historic stone undergoes various degradation processes, including chemical corrosion caused by interactions with metals and pollutants such as sulphur, carbon dioxide, and chlorides [2,3,4,5]. Specifically, copper and its alloys, found in components like clamps, pivots, or plaques [2,6,7], develop a corrosion layer known as patina [8,9]. Exposure to the atmosphere induces oxidation-reduction reactions in copper, resulting in the formation of distinct corrosion products: red copper (I) oxide, black copper (II) oxide, black copper sulphide, and various coloured salts, as well as green-blue compounds like nantokite, atacamite, and clinoatacamite [2,9].

Depending on the location of the metallic elements in contact with the stone material (e.g., screws, structural bars or pedestals, plaques, etc.), the metal corrosion products can either remain on the surface of the artifact or penetrate deeper into the stone. On the surfaces, the most visible consequence of their presence is discoloration [3,6], which causes aesthetic damage to the artifact. However, when salts penetrate the inner layers of stones, much more significant mechanical stresses can occur. These includes increased porosity nand internal capillarity, the formation of cracks, pulverization, and exfoliation of the material. These issues arise due to the increased crystallization pressure of metal salts on the walls of the internal pores [10]. The increased porosity can have various other impacts on the intrinsic properties of the stone material; for example, one of the main elements contributing to the enhancement of bio-receptivity is defined as the totality of material properties that contribute to the establishment, anchorage, and development of fauna and/or flora [11].

To face this issue, several cleaning methods can be considered for an efficient removal of such corrosion layers. As concerns physical methods, laser cleaning is finding numerous applications in removing deterioration products from stone artefacts, such as black crusts, biofilms, and dirty layers [12,13,14]. Researchers are also investigating the possibility of selectively removing Cu-corrosion patinas from copper laminas by Laser-Induced Breakdown Spectroscopy (LIBS) [14]. However, despite many advantages (material selectivity, immediate feedback, etc.), some weak points make the use of laser cleaning complicated. Among these, there are the high costs of the equipment and the use of energetic radiation, which necessitates great caution due to its potential effects on both the operator and the artwork, as it is an irreversible process [15].

Otherwise, traditional techniques for cleaning stone from metal corrosion products involve the application of aqueous solution containing chelating agents [16,17,18,19,20], such as ethylenediaminetetraacetic acid (EDTA) and potassium sodium tartrate (PST or Rochelle salt), which are useful for the selective removal of salts and are active towards a narrow range of ions, especially metal cations [16,21]. However, the use of chelators in aqueous solutions becomes difficult on statues and other vertical surfaces or when long application times are necessary. To overcome these limitations, several valid systems have been developed to confine chelating agents and provide a localized cleaning, as in the case of thickened pastes, nanocellulose hydrogels, clays, pHEMA, gels, gel-like materials, and Highly Viscous Polymeric Dispersions (HVPDs) [19,21,22,23,24,25,26,27,28,29,30,31]. These latter methods (e.g., HVPDs, gels, and gel-like systems) have been chosen here due to the highly encouraging results recently obtained in other research works, particularly for their simplicity in preparation and high level of control in the application [21,28,32,33,34]. When an oscillating shear stress is applied to the surface of a gel, its storage module (G′) remains greater than its loss module (G″) over a wide range of frequencies. Conversely, for HVPDs, a crossover between G′ and G″ curves is almost always present [35]. In these systems, the chelating agents are covalently bonded to the polymer matrix, which enhances the simplicity of the treatment process by preventing chelator deposition on the cleaned surface. This approach is in line with the increasing demand for conservation products that are not only highly effective but also environmentally sustainable and reversible, thereby minimizing any potential secondary effects on treated surfaces [20,21,24,25,26,27,28,29,30,31]. 

One of the widely recognized gel-like systems utilized in conservation practices involves poly(vinyl alcohol) (PVA) and Borax (B) [2,36,37,38,39,40,41,42,43,44], formed through the cross-linking reaction of polyols with boric acid/borate in aqueous media, creating a complex 3D polymeric network. It is important to note that borax, while effective, can be toxic depending on its concentration and exposure conditions. In our study, we incorporated borax (sodium borate) into the gel formulation at concentrations deemed safe for the specific application of cleaning stone surfaces. The borax used is securely fixed within the polymer matrix. The selected concentrations were based on established safety guidelines and relevant literature [45]. The use of borax in conservation and cleaning is carefully regulated to mitigate any potential environmental and health risks. The proposed gel formulations were developed to efficiently remove metal corrosion products while complying with these safety standards. 

Despite the fact that PVA-B systems are characterized by good viscoelastic behaviour, previous studies [2,36,41,46] have shown that they have some limitations when applied on porous surfaces because they may adhere to them and leave residues. To this end, loading the PVA-B system with organic solvents and chelators in variable concentrations, depending on the hydrolysis degree of PVA polymer [26,43], allows one to improve its characteristics. Specifically, it has been demonstrated that the addition of agarose (AG) to the PVA-B network improves its mechanical properties, such as good workability, flexibility, self-healing properties, and no residues left on certain porous materials [36,37]. Indeed, agarose is a non-ionic polysaccharide of high molecular weight. It is extracted from red marine seaweeds and is composed of β-1,3-linked-D-galactose and α-1,4-linked 3,6-anhydro-L-galactose. Agarose undergoes thermoreversible gelation [42]. Agarose, compared to agar, offers higher gel strength and is less prone to degradation over time. This stability is particularly advantageous in the context of our study, where we aimed to evaluate the long-term efficacy of gel-like materials in removing metal corrosion products from stone surfaces. These properties of agarose ensure that the gel maintains its structural integrity and effectiveness throughout the cleaning process, thereby enhancing its practical utility for conservators [47]. While agarose may be more expensive than agar, its enhanced stability and performance justify its use in fundamental studies aimed at advancing conservation practices [48]. Moreover, future comparative studies between PVA-B-agarose gels and PVA-B-agar gels could provide further insight. To the best of our knowledge, only one work on the characterization of a PVA-B system blended with agarose and loaded with EDTA (3%/1% PVA-B/AG  +  0.5% EDTA) is present in the literature [36], whereas no application of this system to the removal of copper corrosion stains from stone has yet been documented. The authors observed that the noticeable increase in the intrinsic storage modulus ($G_{0}$) when EDTA is loaded into the PVA-B/AG network may be ascribed to the formation of hydrogen bonds between some of the EDTA ions and the PVA chains [26]. Moreover, these measurements suggest that the PVA-B hydrogels blended with AG exhibit minor syneresis (e.g., minor stiffer effect), which normally occurs when low polarity solvents or salts (e.g., EDTA) are loaded into the system. The curve of the storage module (G′), for the formulation loaded with 0.5% EDTA, is higher than that of the loss module (G″) all over the frequency range, indicating more gel-like behaviour. Although Berlangieri et al. [21] showed interesting results on the removal of gypsum from carbonatic matrix using potassium sodium tartrate (PST or Rochelle salt), they did not enrich the system with agarose.

This work aimed to assess the cleaning efficacy of copper corrosion products of stones using different formulations of PVA-B systems blended with a small aliquot of agarose and loaded with two different chelators (e.g., EDTA and PST or Rochelle salt [26,36,49]). To achieve this, we utilized non-invasive portable Nuclear Magnetic Resonance (NMR) combined with Scanning Electron Microscopy—Energy Dispersion Spectroscopy (SEM-EDS) and Fourier Transform Infrared Attenuated Total Reflection (FTIR ATR) spectroscopy. Six different gel-like formulations with varying percentages of chelators (EDTA and PST), with or without agarose, were synthesized and analysed using a rheometer to determine their optimal rheological properties.

Three different carbonate stones (Travertine, Lecce stone, and Carrara marble) were artificially stained with copper sulphates. While acknowledging that real-world stone pollution often involves copper oxides, sulphides, and chlorides, our study focused on copper sulphates for practical reasons. Copper sulphates were chosen due to their manageable and controllable characteristics in laboratory settings, ensuring consistent and reproducible contamination processes. Despite their prevalence in urban environments, where sulphate pollution is more common, copper sulphates represent a significant and relevant form of copper corrosion product. The study aims to illustrate the penetration of these copper corrosion products into stone and assess the efficacy of gel formulations with optimal rheological properties for their removal. By concentrating on copper sulphates, we establish a controlled study that serves as a foundational reference for future investigations involving various copper compounds.

The stone surfaces were characterized by SEM-EDS and portable NMR at three stages: before staining, during the presence of copper stains, and after the cleaning process. Portable single-sided NMR was particularly useful for acquiring *T*_2_ relaxation times, which are highly sensitive to the presence of paramagnetic substances like metallic corrosion products, resulting in significant *T*_2_ reduction. This allowed us to effectively monitor the cleaning process and its efficacy.

The SEM-EDS imaging results complemented the NMR findings by analysing the samples at the same three stages. Furthermore, to evaluate the interaction between the gel and the metal corrosion products on the calcareous stones, the gels were analysed before application and after removal from the treated surfaces using an FTIR ATR device. This analysis helped determine whether the cleaning was purely mechanical, due to gel peeling, or if there was any chemical interaction between the gel components and the corrosion products. FTIR spectroscopy was employed to characterize the six gel formulations both before and after their use on the stone surfaces, ensuring a comprehensive understanding of their cleaning efficacy and chemical structure.

## 2. Results and Discussion

### 2.1. Rheological Characterization of the Gel-like Formulations

**Figure 1:**
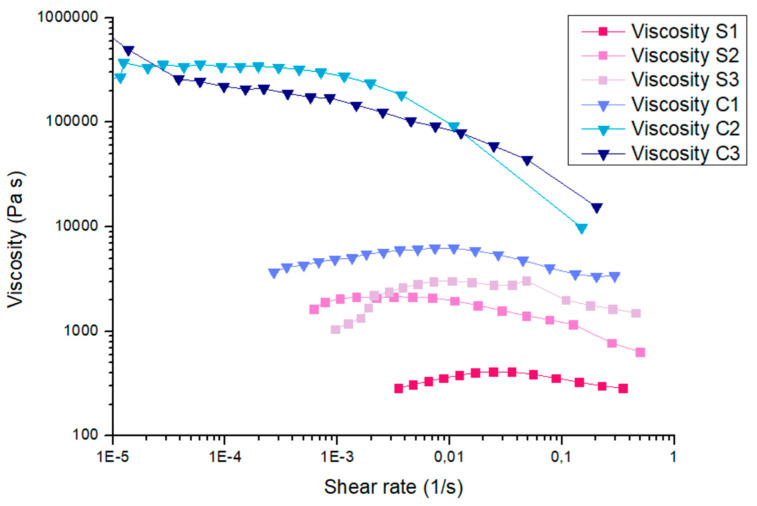
Flow curves of the dynamic viscosity as a function of the shear rate for the six different gel-like formulations.

Figure 1 shows the dynamic viscosity (Pas) as a function of the shear rate (1/s) obtained from flow sweep experiments for the six different soft matter formulations (S1, S2, S3, C1, C2, and C3). 

By looking at the materials’ behaviour, a clear trend can be pointed out. Formulations S2 and S3 exhibited a higher zero-shear viscosity value compared to S1, confirming that the presence of chelators enhances the thickness of the material. The same effect can be observed in the formulations enriched with agarose: samples C2 and C3, containing EDTA and PST, respectively, have a zero-shear viscosity value two orders of magnitude higher than that of C1. Moreover, the viscosity trend for S1, S2, S3, and C1 formulations can be attributed to a pseudo-Newtonian behaviour, since it stays quite stable in the investigated shear rate range. On the other hand, samples C2 and C3 show the typical behaviour of shear-thinning fluids. These are also known as pseudoplastic fluids because their viscosity decreases with increasing shear rate. Specifically, C3 shows a more rapid decrease in viscosity with the shear rate compared to C2.

**Figure 2:**
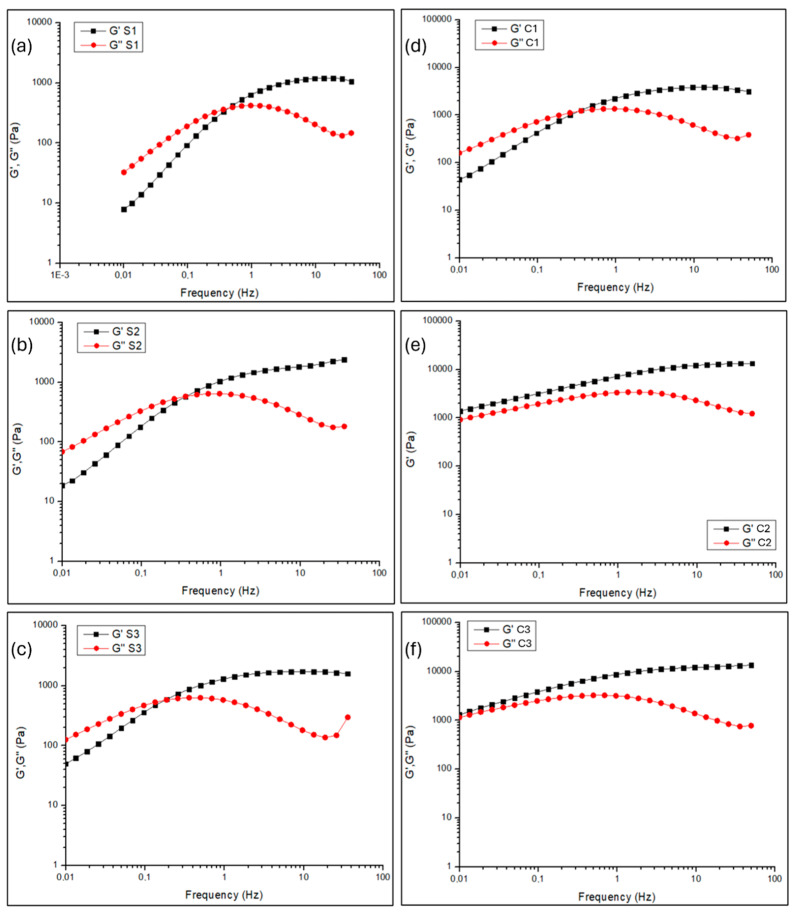
Log-log plot of storage modulus (G′) and loss modulus (G″) versus frequency for samples (**a**) S1, (**b**) S2, (**c**) S3, (**d**) C1, (**e**) C2, and (**f**) C3.

Figure 2 shows the log-log plot of storage or elastic modulus (G′) and loss or viscous modulus (G″) as a function of frequency (ω), useful to study the mechanical properties of the synthesized gel-like formulations. G′ and G″ trends were obtained from frequency sweep experiments in the linear viscosity region (LVR), determined after analyses of strain sweeps. Indeed, frequency sweep experiments are a powerful tool in the rheology of gel-like materials which show the crosslinking behaviour as well as the presence of a reversible network [50]. From a mechanical point of view, the S1, S2, S3, and C1 samples are quite similar, as it can be seen from Figure 2a–d. All samples are frequency-dependent.

In particular, at low frequencies, the samples S1, S2, S3, and C1 are characterized by a loss modulus G″ greater than the storage modulus G′, suggesting a viscous behaviour, until the crossover point, where G″ = G′, is reached. Beyond the crossover point, G′ becomes greater than G″, suggesting an elastic behaviour. The presence of a crossover point was not observed for samples C2 and C3 (Figure 2e,f), which are also characterized by higher G′ and G″ moduli compared to the other samples. A crossover frequency ($\omega_{c}$) indicates reversible cross-linking, whereas the lack of a crossover frequency indicates permanent chemical cross-linking [50]. For samples S1 and S2, the crossover frequency is around 0.5 Hz, while samples S3 and C1 have a crossover point at lower frequencies of around 0.26 Hz and 0.36 Hz, respectively. The apparent relaxation time ($\tau_{C}$), calculated as $2\pi /\omega_{c}$, is around 24 s for the S3 sample, 17 s for the C1 sample, and 13 s for S1 and S2 samples. The increase in $\tau_{C}$ for S3 and C1 samples compared to S1 and S2 samples accounts for better shape stability [21,36]. The G′ and G″ moduli at the crossover point are significantly greater for the C1 sample. These results agree with the literature [50], according to which the addition of fillers to the gel-like formulation shifts the crossover frequency to lower frequencies and higher moduli. In our case, the addition of 1% of PST chelator in the S3 sample shifted its crossover frequency ($\omega_{c}$) to a lower value, and the addition of 0.5% of agarose to the C1 sample both lowered its crossover frequency and increased its G′ and G″ moduli at the crossover point. The apparent relaxation time suggests that the S3 sample, made of 1% PST, is the formulation with the most stable shape. The situation is different for the C2 and C3 formulations, where the addition of agarose and chelators, EDTA, and PST completely modified the mechanical properties of the system that also contains agarose. The elastic modulus G′, which is related to the rigidity of the system [51], is always greater than the viscous modulus G″ and it increases with increasing frequency. C2 and C3 formulations exhibit a more rigid and elastic structure with a more gel-like behaviour [21,36]. Indeed, previous works [36,42] suggested that the inclusion of organic liquids (such as agarose, EDTA, and PST) in PVA-B systems strengthens the gel-like network, as the organic solvents stimulate the boron ions to bind to the PVA chains. The result is a stiffer gel-like material, and this could be attributed to the increased physical interactions within the network [26,52,53].

**Figure 3:**
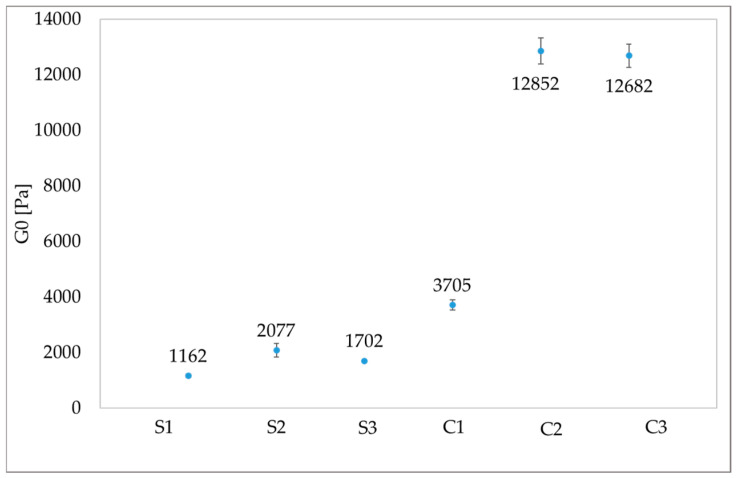
Intrinsic elastic modulus $G_{0}$ of each gel-like formulation.

In Figure 3, the intrinsic elastic modulus $G_{0}$, which is related to the stiffness of the material, calculated from the average of the last five values in the plateau region, is displayed for each sample. The increase in the elastic modulus $G_{0}$ in C1, C2, and C3 samples loaded with agarose, EDTA, and PST indicates the structuring effect on the system of the additives, such as the formation of hydrogen bonds among some ions of AG, EDTA, and PST and the PVA chains in the PVA-B system [36]. Moreover, it has been shown that a soft matter should preserve adequate elastic properties, even upon the addition of additives, and should have a $G_{0}$ > 400 Pa [21,54] to be easily removed by peeling without leaving residues on the treated substrates. To this end, Figure 3 suggests that all samples are characterized by values of $G_{0}$ > 400 Pa and therefore should be suitable to be removed by peeling, leaving no residue. Among all the formulations, C2 and C3 are the only ones exhibiting a more gel-like behaviour (G′ always higher than G″), with the highest *G*_0_ values that suggest an increased elasticity even upon addition of agarose and chelators. It is worth noting that the S2 sample, in which EDTA was added to the PVA-B network, preserves almost the same properties (e.g., same $\omega_{c}$ and $\tau_{C}$) as the S1 sample, to which no filler was added. Only a small increase in the intrinsic elastic modulus $G_{0}$ was observed in the S2 sample. Differently, when PST was added to the S3 formulation, an increase in both $G_{0}$ and $\tau_{C}$ along with a lowering of the $\omega_{c}$ were registered. This result indicates that EDTA and PST have different behaviour in the PVA-B network. According to Berlangieri et al. [21], EDTA content is lower than the limit of 0.5%, which corresponds to the maximum concentration after which the mechanical properties of the gel-like material start to change. Conversely, PST is over the 0.9% limit suggested by Berlangieri et al. [21], and therefore the addition of this chelator has significantly modified the mechanical properties of the PVA-B system, which exhibits a more gel-like behaviour.

### 2.2. NMR and SEM-EDS Monitoring of Untreated and Stained Carbonate Stones

**Figure 4:**
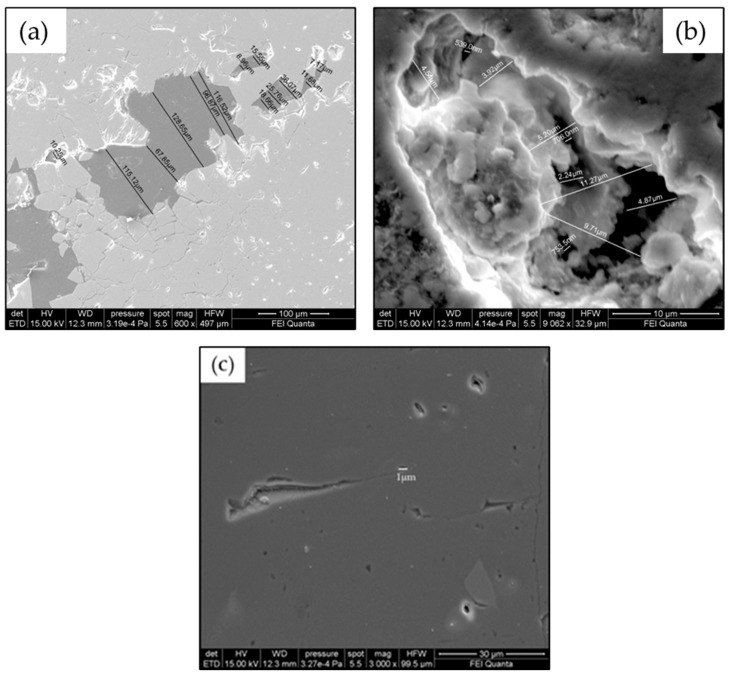
SEM images of untreated (**a**) Travertine, (**b**) Lecce stone, (**c**) Carrara marble.

**Figure 5:**
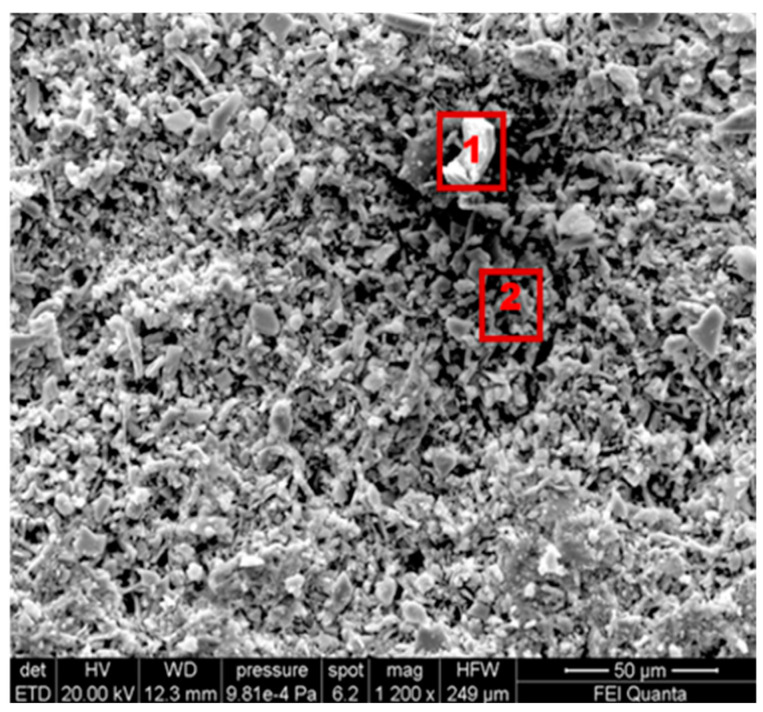
SEM image of the Travertine surface covered with a copper sulphate layer.

**Figure 6:**
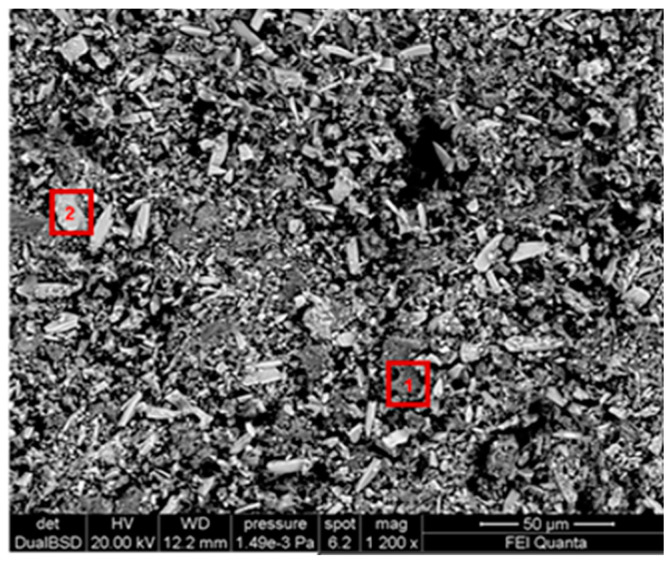
SEM image of the Lecce stone surface covered with a copper sulphate layer.

**Figure 7:**
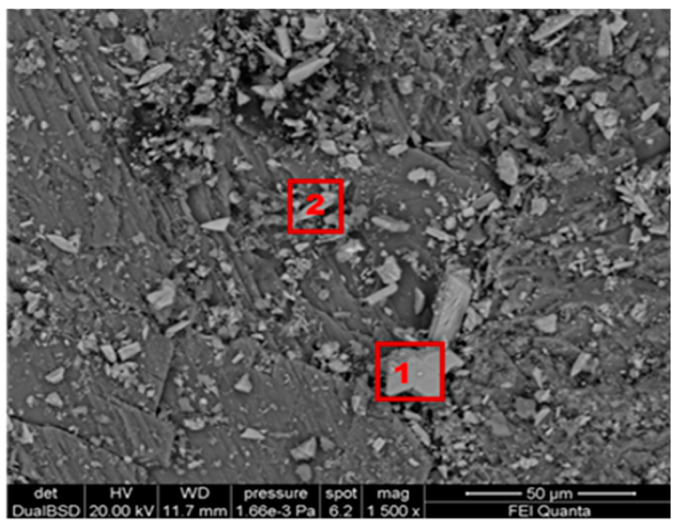
SEM image of the Carrara marble surface covered with a copper sulphate layer.

Figure 4 shows the morphological SEM images obtained from Travertine, Lecce stone, and Carrara marble before contamination with metal corrosion products. Travertine is characterized by a macroporosity, directly measured on these images, with a mean pore diameter around 60 μm. Lecce stone presents a mesoporous structure characterized by pores with diameters between 1 μm and 10 μm. Carrara marble shows a microporous structure made of pores with diameters between 0.1 μm and 1 μm, as is well known from the literature [55,56,57,58,59,60,61]. SEM images collected from the contaminated surface of each sample of Travertine, Lecce stone, and Carrara marble are shown, respectively, in Figure 5, Figure 6 and Figure 7. The EDS spectra are reported in the Supplementary Materials, under the names: Figure S1 EDS spectra of Figure 5, from points 1 and 2 on stained Travertine with copper sulphate layer, Figure S2 EDS spectra of Figure 6, from points 1 and 2 on stained Lecce stone with copper sulphate layer, Figure S3 EDS spectra of Figure 7, from points 1 and 2 on stained Marble stone with copper sulphate layer.

SEM superficial images show a clear distribution of crystals with irregular shapes on the stone surfaces. The EDS spectra determined the chemical nature of these crystals and are reported in Supplementary Materials. Since all samples studied are carbonate stones mostly made of calcium carbonate (CaCO_3_) and very low amount of impurities [58,59,60,62,63,64], the EDS peaks associated with the corrosion layer are mainly those of copper (Cu) and sulphur (S). This result indicates that the copper corrosion layer present on the surface of all samples is composed of crystals of hydrated copper sulphate. It is worth noting that the copper sulphate crystals seem more abundant on the Lecce stone surface. 

**Figure 8:**
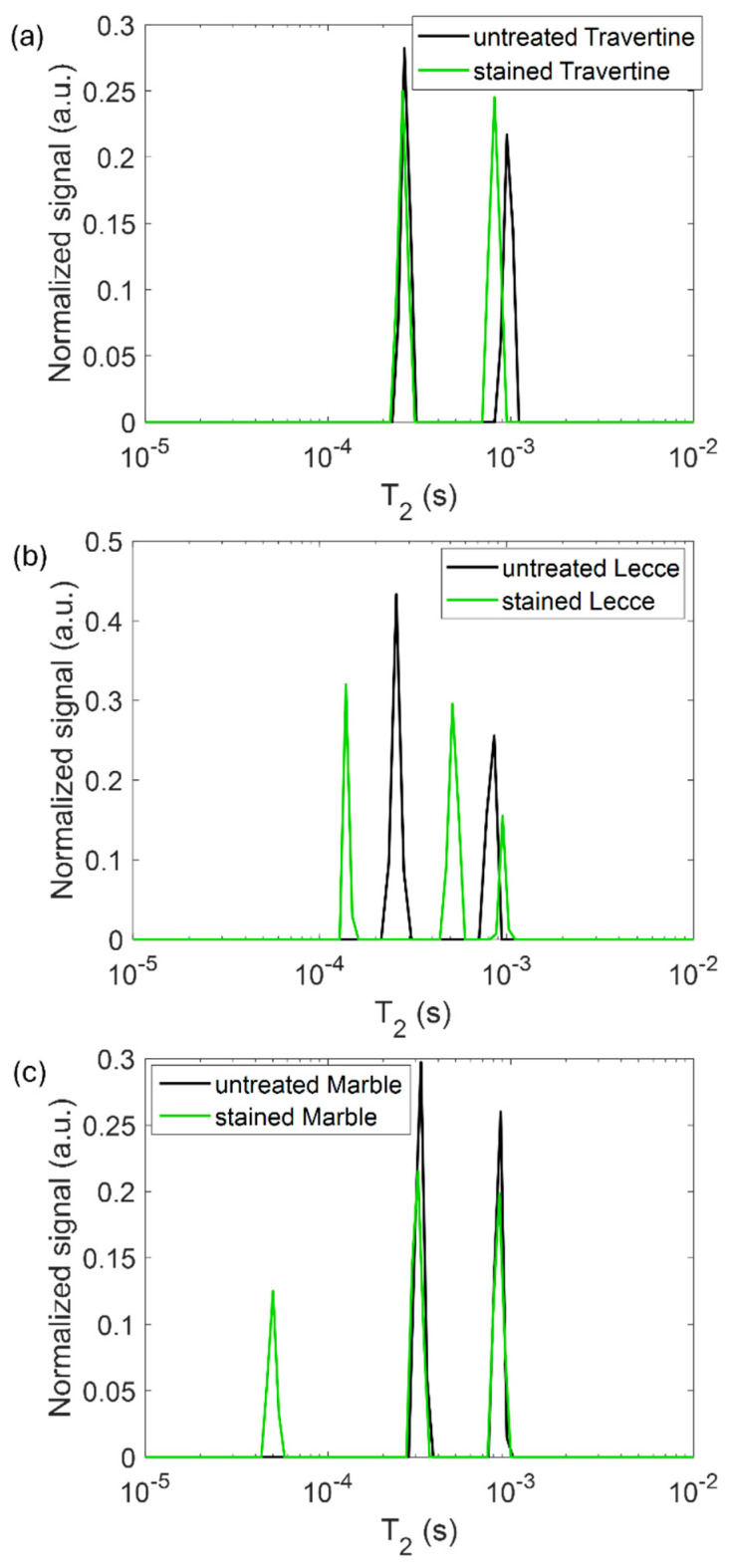
*T*_2_ relaxation times distribution for (**a**) Travertine, (**b**) Lecce stone, and (**c**) Carrara marble before (black line) and after (green line) the contamination process.

In Figure 8, the spin-spin relaxation time T_2_ distribution measured on the untreated samples (black line) and the samples contaminated with metal corrosion products (green line) is displayed. We performed NMR measurements following previous works [32] on the stained samples to test if the *T*_2_ parameter was affected by the presence of metal corrosion products and if it could inform us about different degrees of contamination of the samples. For each sample, two different *T*_2_ components were detected. The long *T*_2_ component is associated with water molecules that have a weaker interaction with the pores walls, whereas the short *T*_2_ with water molecules that strongly interacts with the pore walls [2,32]. A general decrease in the *T*_2_ value was obfserved for all samples. This result confirms the sensitivity of the spin-spin relaxation time to paramagnetic substances [2] (e.g., salts and metals) deposited on the stone surface. For Lecce stone and Carra marble, even a third *T*_2_ component was detected, indicating the formation of a new population of hydrogens attributable to the corrosion products. The shortening effect of paramagnetic substances on *T*_2_ is sharper in Lecce stone, where all the *T*_2_ peaks are far from those measured on the untreated sample. The contamination effect seems instead weaker for Travertine and marble. This result suggests a different staining degree and penetration of corrosion in the samples.

### 2.3. Visual Inspection of the Cleaning Ability of the Gel-like Formulations

**Figure 9:**
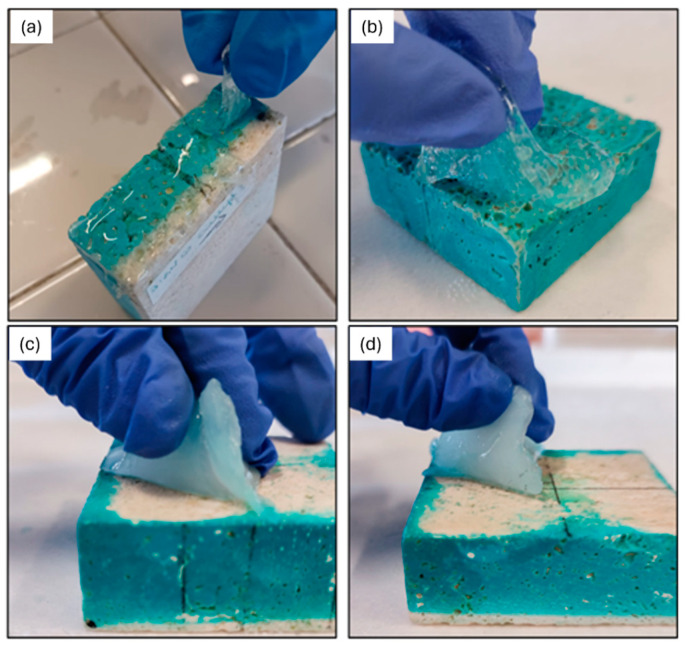
Peeling process after 4 h of drying of the gel-like formulations enriched with (**a**) EDTA, (**b**) PST, (**c**) AG and EDTA, (**d**) AG and PST.

**Figure 10:**
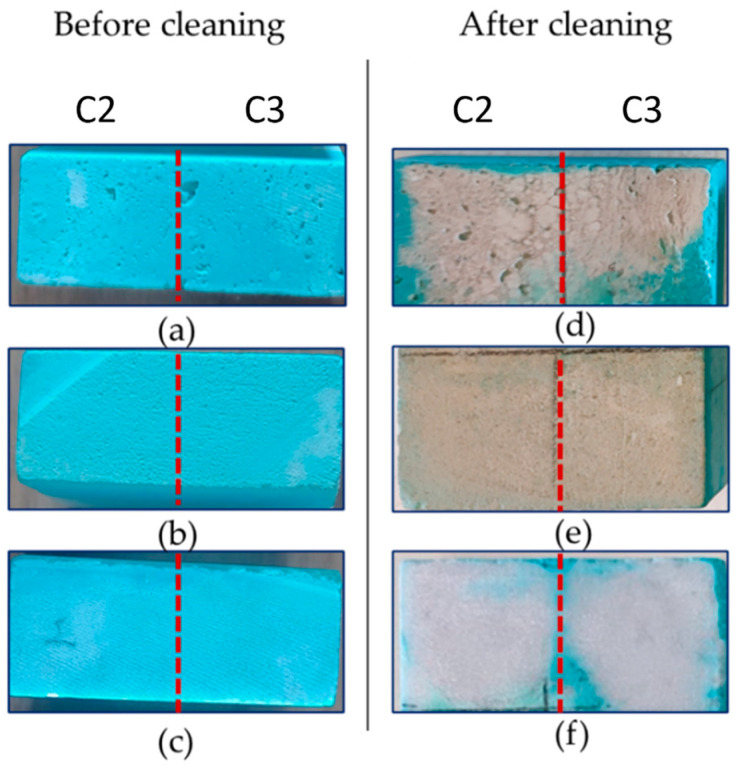
Surface of (**a**,**d**) Travertine, (**b**,**e**) Lecce stone, and (**c**,**f**) Carrara marble before cleaning and after two cleaning processes. Stone samples were treated with C2 formulation to the left of the red line and with C3 formulation to the right of the red line.

Gel-like formulations enriched with EDTA or PST, with (C2 and C3 formulations) and without agarose (S2 and S3 formulations), were used to clean the stone surfaces following the procedure explained in Section 4.4. Figure 9 shows that among the four formulations, S2 and S3 are less easy to apply and to peel off. They also left residues because of their weaker elasticity. Conversely, C2 and C3 samples show adequate elasticity and easier application and peeling (Figure 9c,d). For this reason, from now on, the S2 and S3 formulations are excluded from further tests. From a first visual investigation, the two formulations enriched with agarose and chelators (C2 and C3) provided an incomplete removal of the blue layer of copper sulphate. For this reason, a second cleaning test was performed 24 h after the first. Figure 10 shows the stone surfaces before and after the two application steps of C2 and C3 formulations. A clear difference between zones where gel-like formulations were applied and those not treated is seen. 

### 2.4. SEM and NMR Monitoring of the Gel-Cleaned Stones

**Figure 11:**
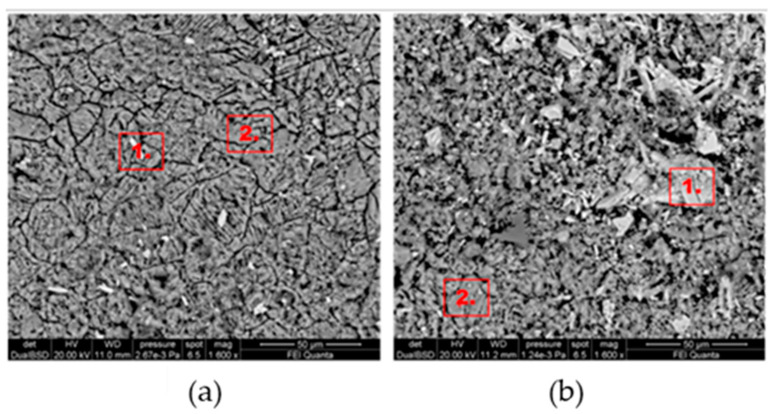
SEM images Travertine cleaned with (**a**) C2 formulation and (**b**) C3 formulation.

**Figure 12:**
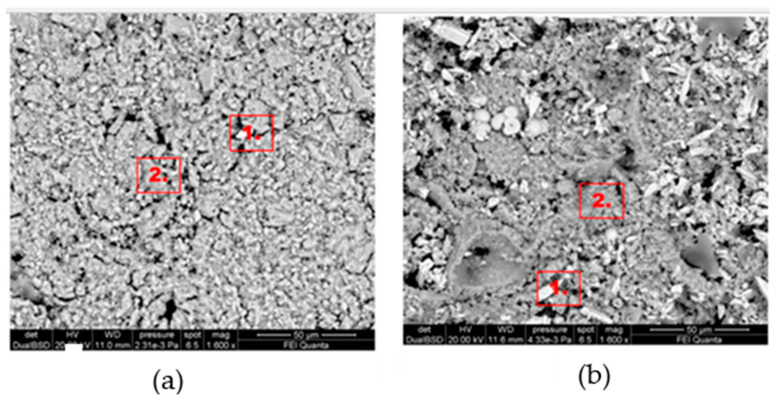
SEM images of Lecce stone cleaned with (**a**) C2 formulation and (**b**) C3 formulation.

**Figure 13:**
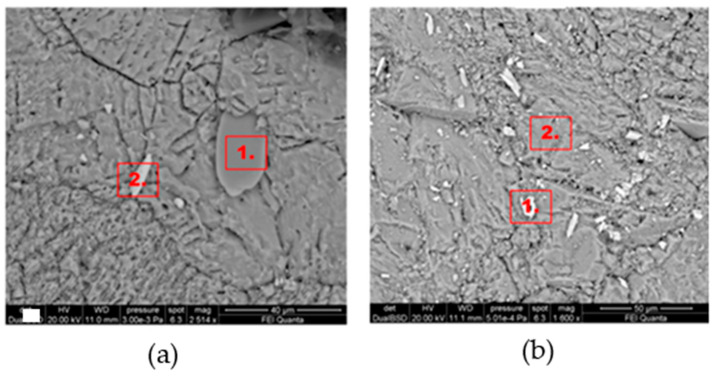
SEM images of Carrara marble cleaned with (**a**) C2 formulation and (**b**) C3 formulation.

Figure 11, Figure 12 and Figure 13 show the SEM images (EDS spectra are reported in the Supplementary Materials, under the names Figures S4–S6) of Travertine, Lecce stone and Carrara marble, respectively, cleaned with C2 and C3 formulations. It can be seen that almost the entire layer of copper sulphate was removed with the gel-cleaning. However, the chemical composition obtained from EDS spectra still suggests the presence of copper sulphate residues, which can be identified as lighter crystals on a darker background. Furthermore, the EDS spectra did not detect any elements belonging to the chemical composition of the gel-like formulations, indicating that both C2 and C3 samples did not leave residues, guaranteeing the reversibility of the products. Qualitatively, it seems that the best results were obtained on the surfaces treated with the C2 formulation, as can be deduced from the presence of fewer residual copper sulphate crystals in the SEM images. 

**Figure 14:**
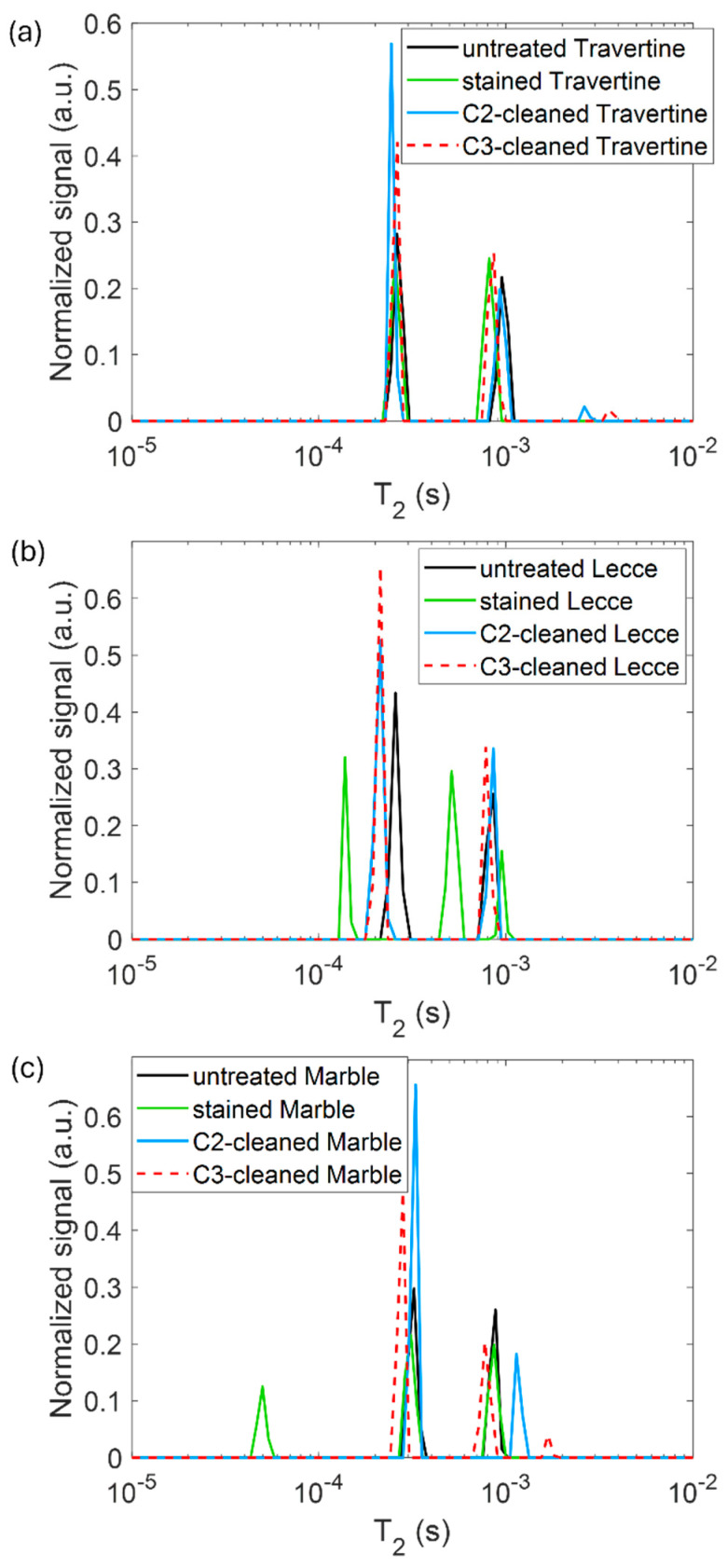
*T*_2_ distribution measured on the untreated, stained, and soft matters-cleaned surface of (**a**) Travertine, (**b**) Lecce stone, and (**c**) Carrara marble.

The relaxation time *T*_2_ distribution, acquired after the cleaning process with C2 (light-blue line) and C3 (red dashed line) formulations on the samples of Travertine, Lecce stone and Carrara marble, is shown in Figure 14. 

After cleaning, the *T*_2_ measured on all samples partially returned to the original values measured on the untreated samples. For Travertine cleaned with C2, both the short and long *T*_2_ components switched back to their original value, whereas when it was cleaned with C3, the long *T*_2_ component still remained equal to that measured on the stained surface (green line). This indicates that in the case of Travertine, the C2 formulation worked considerably better than the C3. Lecce stone, which was the sample with stronger contamination (as shown in Figure 6 and Figure 8), shows that both C2 and C3 formulations behave similarly. Both the short and long *T*_2_ components only neared their original value, indicating a worse cleaning of this stone surface. Good results were obtained for Carrara marble with both C2 and C3 formulations. However, when marble was treated with the C2 formulation, the original short *T*_2_ component was completely restored, whereas the long *T*_2_ component increased. Differently, when treated with C3, both the *T*_2_ components only got closer to their original value. Overall, from these NMR measurement, it seems that the gel-like formulation containing EDTA and agarose works better than that containing PST and agarose, in agreement with the SEM-EDS result. The best cleaning action was achieved for Travertine, whereas the worst was found for Lecce stone. For the latter, we can hypothesize that the cause of the worse cleaning effect is the high quantity of the corrosion products, which would require a further cleaning step to be removed. Overall, the incomplete restoration of the original *T*_2_ values of all stone samples after cleaning, except for Travertine cleaned with C2 formulation, indicates that metal corrosion products were not completely removed [2], in good agreement with SEM-EDS results.

### 2.5. FTIR Characterization of the Gel-like Formulations

**Figure 15:**
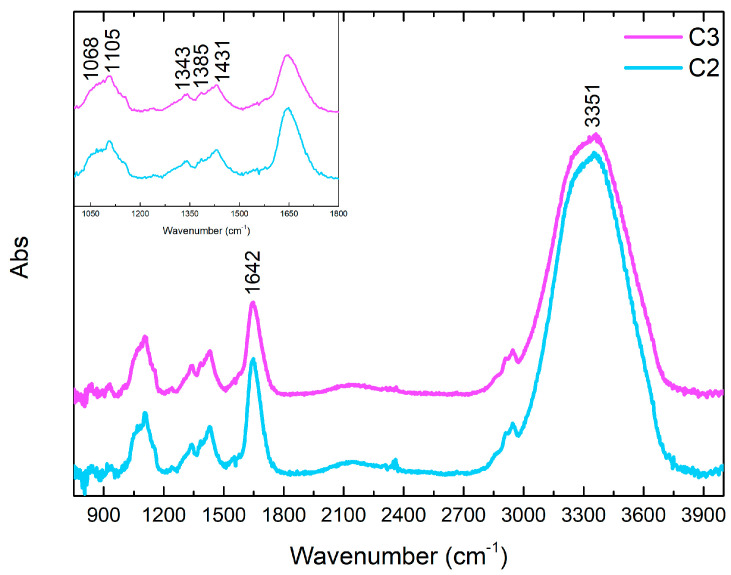
FTIR spectra of C2 and C3 gel-like formulations.

Figure 15 shows the FTIR characterization of the six different gel-like formulations. The ATR FTIR spectrum of PVA-B system shows a general correspondence with the spectra of similar systems reported in the literature [36,65,66]. The visible peak at 1088 cm^−1^ is attributable to PVA C-O stretching, while the signals at 1237 and 1386 cm^−1^ (shoulder) are indicative of CH_2_ wagging modes [36]. The bands at 1339 and 1431 cm^−1^ are attributed to the tetrahedral and trigonal, respectively, complex-crosslinking of PVA with borate (B-O-C stretching relaxation), while the barely visible shoulders at 1319 and 1419 cm^−1^ could be related to the pure C-O-H and CH_2_ bending, respectively, in non-crosslinked PVA portions [36,65,66]. The bands at 1648 and 3358 cm^−1^ are indicative of OH bending and stretching, respectively, while the low-intensity signals at 2925 and 2944 cm^−1^ are attributable to CH_2_ stretching [36]. It is interesting to notice that, in the case of the addition of EDTA in S2 formulation, the spectrum shows a general variation of the signal between 1000 cm^−1^ and 1185 cm^−1^, with the splitting of the band at 1088 cm^−1^ in a shoulder at 1068 cm^−1^ and a peak at 1105 cm^−1^ as well as the disappearance of the peak at 1239 cm^−1^ and a general low decrease in the intensity of signals of OH groups. These variations could be indicative of a chemical interaction between the EDTA and the PVA/B system [26]. The addition of PST in S3, instead, does not involve great variations in the spectral pattern of the polymer formulation, except for the remarkable increase in OH signals at 1648 and 3358 cm^−1^ and of C-O stretching at 1090 cm^−1^, attributable to the corresponding groups of the PST. Moreover, an intense peak at 893 cm^−1^ appears, probably indicative of C-C bending of PST [67]. From these data, it is possible to hypothesize that the addition of EDTA would correspond to a stronger chemical interaction towards the PVA-B system, in comparison to the analogue addition of PST, as already observed by the rheological properties (see Section 2.1). About the other formulations, the addition of agarose in C1 is observable, in the related spectrum, from signals between 1010 and 1170 cm^−1^: the absorption shoulder around 1148 cm^−1^ is indicative of the formation of H-bonds and crystallinity of PVA, while a small peak at 1068 cm^−1^ could be attributed to the C-O-C glycosidic bond [36]. The peak of PVA C-O stretching is observable, in this case, at 1105 cm^−1^. The absorption around 1300 cm^−1^ and the intensities of C-H stretching find a correspondence in the literature, as representative of the sum of the two components [36]. Finally, it is also interesting to notice that the peak at 1239 cm^−1^ is almost not observable. The spectra of C2 and C3 formulations show a general similarity with that from the PVA-B/AG system, but it is important to highlight that all the peaks at 1105, 1343, 1385, and 1431 cm^−1^ are subjected to a slight increase in intensity and they develop a more defined distribution. Taking into account the fact that they are attributed to the PVA interactions with the agarose (1105 cm^−1^) and with the borate ions (1343 and 1431 cm^−1^), a chemical effect on the cross-linking of the polymer attributable to the chelators can be hypothesized.

FTIR study of S2, S3, S1, and C1 formulations before being used are in the Supplementary Materials, under the names Figure S7 FTIR spectra of S2 and S3 formulations before being used, and Figure S8 FTIR spectra of S1 and C1 formulations before being used.

#### FTIR Study of C2 and C3 Formulations before and after Cleaning

**Figure 16:**
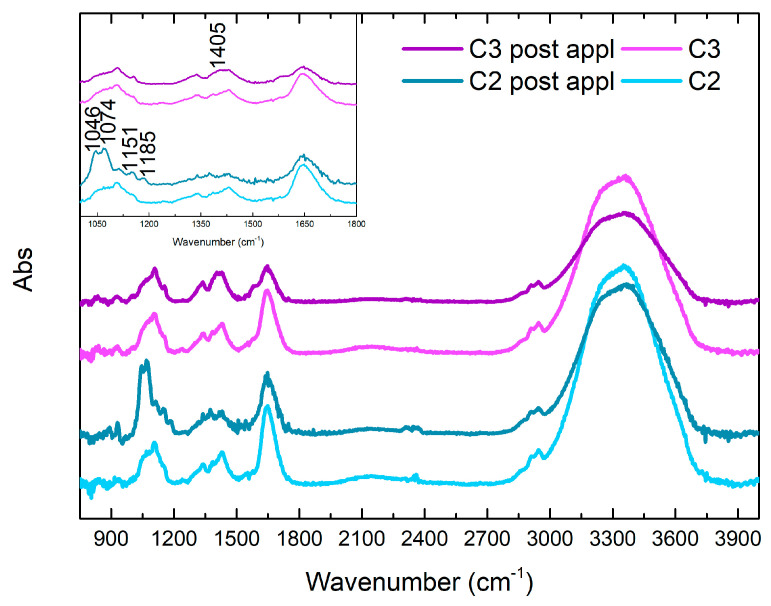
FTIR spectra of C2 and C3 formulations before and after being used to clean Travertine.

**Figure 17:**
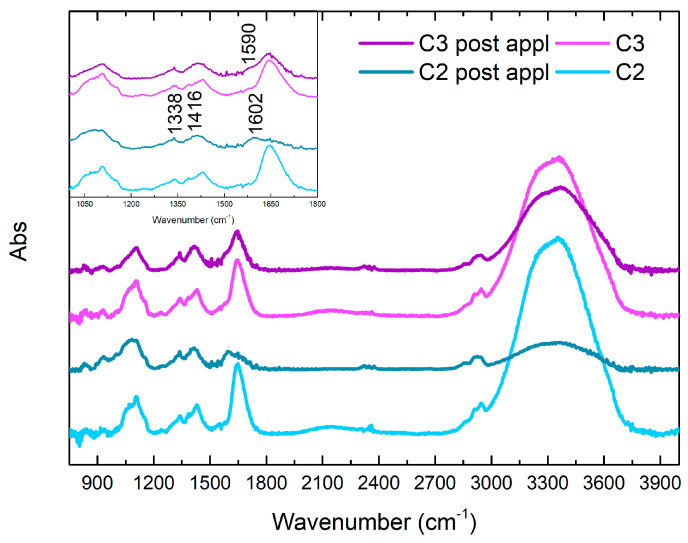
FTIR spectra of C2 and C3 formulations before and after being used to clean Lecce Stone.

**Figure 18:**
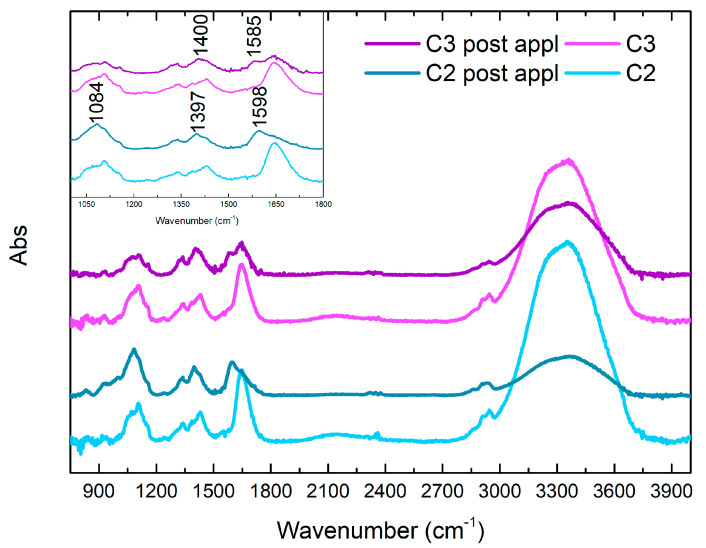
FTIR spectra of C2 and C3 formulations before and after being used to clean Marble.

FTIR analysis, shown in Figure 16, Figure 17 and Figure 18, was used to evaluate the cleaning mechanism of the C2 and C3 formulations on the stone substrates. A general difference in the FTIR signals could be observed concerning the C1 analogue and the application substrate. For the Travertine sample, both the samples show only a limited number of variations attributable to the interaction with the substrates: for C3, the only observed difference is a decrease in intensity of OH signals at 1648 and 3361 cm^−1^ and the appearance of absorption at 1405 cm^−1^; in the case of C2, signals with higher intensity are observable at 1046, 1058, 1074, 1113, 1151, and 1187 cm^−1^. Some of these signals could be attributed to the presence of copper sulphates with different hydration ratios (1058, 1074 cm^−1^) [68], while for the other signals, a certain attribution is not straightforward, but a similarity could be observed with signals of calcium sulphates [69]. Finally, the shoulder at 1118 cm^−1^ could be indicative of a direct complexation of the polymer with the copper ions [70]. In general, these new signals and the absence of main variations related to the polymer-chelator formulation suggest that the C2 interaction with the degradation layer on the surface of the Travertine is probably mostly mechanical, while the removal of the copper corrosion products is less efficient for the C3 formulation. In the case of the Lecce stone, the C2 spectrum shows a new absorption at 1001 cm^−1^, while the peak at 1108 cm^−1^ disappears. On the contrary, it is interesting to notice that there is a great decrease in intensity of the OH bands, while a signal with a clear maximum at 1601 cm^−1^ appears. This variation is indicative of the complexation of EDTA with Cu(II), which presents a covalent character [71]. Another interesting variation is related to the shift of the signal at 1429 cm^−1^ to 1415 cm^−1^, with a decrease in intensity, which could correspond to an interaction of borate ions with the copper ions, along with the sharper definition of the signal at 1339 cm^−1^. On the contrary, for the C3 formulation, the major variations are represented by a general decrease in the intensity of the OH signal, while only a slight shoulder is observable around 1590 cm^−1^, attributable to PST [68,72,73] interaction with the metal. The variations for the signals at 1339 and 1429 cm^−1^, indicative of borate stretching, are instead observable, even if generally less evident. These data would be indicative of a higher interaction with copper ions through the polymer moieties than through the chelator groups. Finally, in the case of Carrara marble, spectral differences before and after the application on the substrate become clearly visible for both the formulations. For C2, the complexation between the EDTA and the copper ions is confirmed by the signal at 1598 cm^−1^ (which, in this case, presents higher intensity than the OH peak at 1648 cm^−1^), while the interaction through the borate is confirmed by the shift of the peak from 1430 to 1397 cm^−1^. Moreover, for this sample, it is interesting to notice that the PVA C-O stretching peak at 1105 cm^−1^ shifts to 1084 cm^−1^ with a general broadening: this could imply that some bonds between the two polymeric species are lost after the interaction with the corrosion products on the surface of the substrates. The new interaction between the EDTA and metal ions would involve the breaking of previous non-covalent cross-linking between the chelator and PVA, while a series of overlapping signals related to C-O-Cu interactions would be formed in this region [71]. Similar results are observed for the case of the application of C3 on the marble substrate, with the formation of a new band at 1587 cm^−1^ attributable to PST–copper interactions [72,73], a shift from 1430 to 1405 cm^−1^ for borate stretching, and a more intense but broader band around 1083 cm^−1^, even if they can be considered generally less intense than for the C2 analogue. With reference to these results, it is possible to state that the application of C2 for cleaning has better performances than C3, confirming the data from previous analyses. Moreover, the cleaning mechanism changes from mostly mechanical to chemical when varying the substrate from Travertine to Lecce stone to Carrara marble.

## 3. Conclusions

This study is a proof of concept to evaluate the cleaning efficacy of PVA-borax based systems to remove copper corrosion layers made of sulphates from stones. Toward this goal, the study proposes a multi-technique protocol based on portable NMR non-invasive measurements, SEM-EDS analysis, and FTIR spectroscopy to evaluate the removal of copper sulphates from stones using modified PVA-borax systems enriched with agarose and loaded with two chelating agents: EDTA and PST.

The addition of agarose and chelating agents to the PVA-B network produces a gel-like behaviour with greater elasticity and ease of application and removal. The increased rigidity of these formulations allows for the application of a gel-like material layer on stones with micro-, meso-, and macroporosity without leaving residues inside the pores after removal.

To assess the cleaning effectiveness on lithotypes, portable NMR non-invasive measurements and SEM-EDS analysis were used. The portable NMR identified the best cleaning performance non-invasively, detecting various levels of metal contamination and different cleaning degrees of the stones. The NMR results were corroborated by SEM-EDS analyses, which also identified the corrosion products. 

FTIR spectroscopy was used to analyse the gels before and after application, providing crucial information on the chemical composition and changes in the gels during the cleaning process. FTIR spectroscopy revealed that the cleaning mechanism depends on the stone’s porosity: it is mainly mechanical for macroporous stones and more chemical for meso- and microporous stones, where both EDTA and borate interact with the copper ions of the sulphates.

This combined approach suggested that the most suitable formulation for removing the copper stains from stones is the one containing 0.5% agarose and 1.5% EDTA incorporated into the PVA-B network (formulation C2). After two consecutive applications of 4 h each, the AG-EDTA-PVA-B system seems to remove most of the metal corrosion products, leaving only minor traces of copper sulphate crystals on all stones with different porosities. However, the best performance is achieved when the cleaning mechanism is mechanical on the macroporous surface of Travertine.

This work highlights the great potential of portable NMR as a non-invasive and non-destructive technique for evaluating cleaning processes on lithotypes. It also emphasizes the necessity of adding agarose to make the system removable by peeling. For future work, we will consider using portable NMR and the gel-like formulation containing EDTA and agarose for an extended application time (more than 8 h), divided into several 4 h steps, to verify if even minor traces of metal can be removed from the stone surface.

Moving ahead, potential future directions arising from this study include exploring the application of our optimal gel formulation to copper-contaminated stone fragments. This step aims to assess the practical effectiveness of the gel in real-world scenarios of copper contamination on stone surfaces. Additionally, further investigation into the interaction mechanisms between the gel and copper corrosion products is warranted. This research pathway seeks to potentially refine gel formulations and explore their efficacy in the cleaning and preservation of stone materials, which could contribute to advancements in cultural heritage conservation practices.

## 4. Materials and Methods

### 4.1. Materials

**Figure 19:**
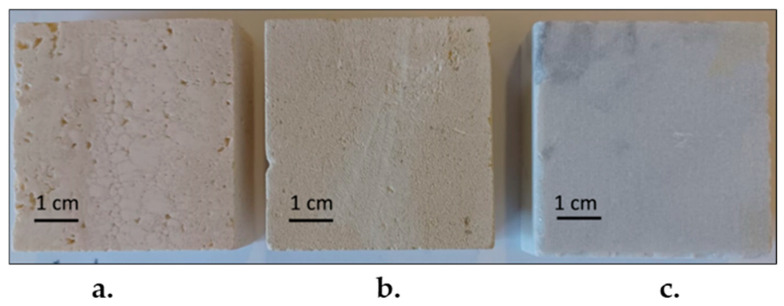
Samples of (**a**) Travertine, (**b**) Lecce Stone, and (**c**) Carrara marble used in this work.

Three carbonate stones with different physical properties (in terms of porosity and capillarity) belonging to three different lithotypes, Travertine, Lecce Stone, and Carrara Marble, were studied in this work (Figure 19). They were chosen because they are among the most representative stone materials in the field of cultural heritage. Three samples of each lithotype with a parallelepiped-like shape and size of 5 × 5 × 2 cm^3^ were purchased from STILMARMI (Casalvieri, Italy). Moreover, three additional Travertine samples of the same size were used to conduct preliminary tests. 

Two bronze disks type 555, with nominal composition of 85% Cu, 5% Sn, 5% Pb, and 5% Zn, produced by the Institute for the Study of Nanostructured Materials, National Research Council (ISMN-CNR), along with pure anhydrous Copper (II) Sulphate (CuSO_4_), supplied by AppliChem (Ottoweg, Germany), were used to trigger the artificial deposition of the copper sulphates on the three stone samples.

Two types of polyvinyl alcohol (PVA) supplied by Sigma Aldrich (St. Louis, MO, USA) were used to synthetize two different set of gel-like materials: PVA with molecular weight (Mw) = 47,300 kDa and hydrolyzation degree = 80% and PVA with Mw = 146,000–186,000 kDa and hydrolyzation degree = 98–98.8%. Di-Sodium tetra-Borate 10-hydrate (Borax, B) was supplied by AppliChem (Germany). The gel-like formulations were loaded with potassium sodium tartrate 4-hydrate (PST or Rochelle salt), ethylenediaminetet-87 tetraacetic acid (EDTA) supplied by AppliChem (Germany), and Agarose (AG) supplied by Euroclone (Pero, Italy). All reagents were used as received.

### 4.2. Synthesis of the Gel-like Formulations

In Table 1, the different formulations of the six gel-like materials, synthesized starting from the procedure found in the literature [26,36,37,42] but with small modifications, are shown. The synthesis procedure was performed at room temperature, with only the aid of a magnetic stirrer on a heating plate. Two stock solutions in distilled water were obtained: one containing 6% *w*/*w* of borax (B) and one 4% *w*/*w* of PVA. According to previous recipes found in the literature [37,44,74], two types of PVA with different molecular weights were employed in the gel-like formulations in order to improve their mechanical properties, elasticity, and peeling characteristics. The PVA with higher molecular weight was used in the formulations without agarose, while PVA with lower molecular weight was used in the formulations with agarose. It is worth noting that to obtain the complete dissolution of PVA in water, it was necessary to heat the solution at 90 °C, under constant magnetic stirring, until a homogeneous and clear mixture was obtained. The two stock solutions were then mixed in a ratio of PVA:B = 4:1. 

**Figure 20:**
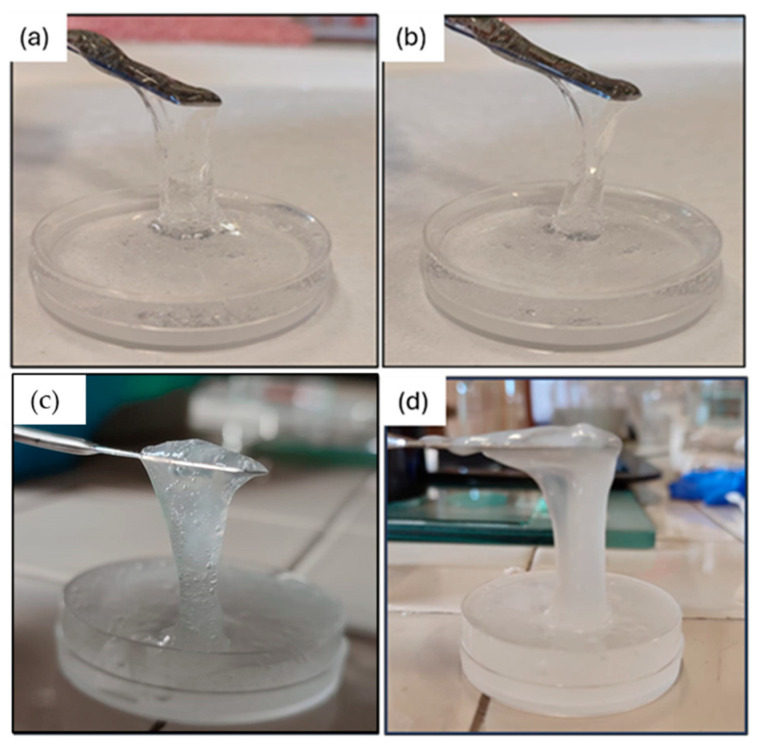
The appearance of the PVA-B system loaded with (**a**) EDTA, (**b**) PST, (**c**) agarose and EDTA, and (**d**) agarose and PST.

This mixture represents the basic PVA-B network, named as S1, from which all the other formulations have been obtained according to Table 1. Among these six formulations, S2, S3, C2, and C3 (shown in Figure 20) are those loaded with chelators (e.g., EDTA and PST), and therefore they were used for cleaning tests.

### 4.3. Rheological Characterization of the Gel-like Materials

The mechanical properties of the different gel-like formulations were characterized from a rheological point of view by flow sweep and frequency sweep experiments to understand their behaviour during application and removal by peeling from the stone surface. Approximately 800 μL of each gel-like formulation was allowed to stabilize at T = 25 °C in the thermostatic room of the rheometer for 2 h before being analysed. A stress-controlled rheometer, specifically a TA Discovery HR1 model, operated by TRIOS software version 4.3.0.38388 was used. The employed geometry was plate-plate, serrated, and made of aluminium, with a diameter of 20 mm. The operational gap was set at 500 µm.

### 4.4. Artificial Contamination and Cleaning Tests of Carbonates Samples

**Figure 21:**
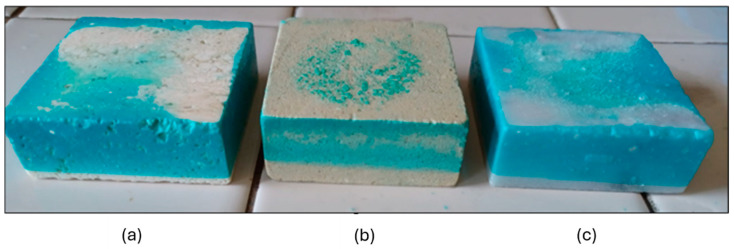
(**a**) Travertine, (**b**) Lecce stone, and (**c**) Carrara marble contaminated with metal corrosion products resulting from the corrosion of two bronze disks in an aqueous solution of CuSO_4_.

Following the method described by Di Carlo et al. [74], two bronze disks were immersed for 2 h under magnetic stirring in an aqueous solution (distilled water) of CuSO_4_ 17 mM. Then, the two bronze disks were moved into a second aqueous solution of CuSO_4_ 50 mM where they were left for 15 days at room temperature. From this process, a final solution containing soluble metal salts was obtained. The Travertine, Lecce Stone, and Carrara marble samples were immersed up to 1.5 cm in height. The samples remained in this solution for 5 days and then were left to dry at room temperature for 2 weeks (see Figure 21). 

**Figure 22:**
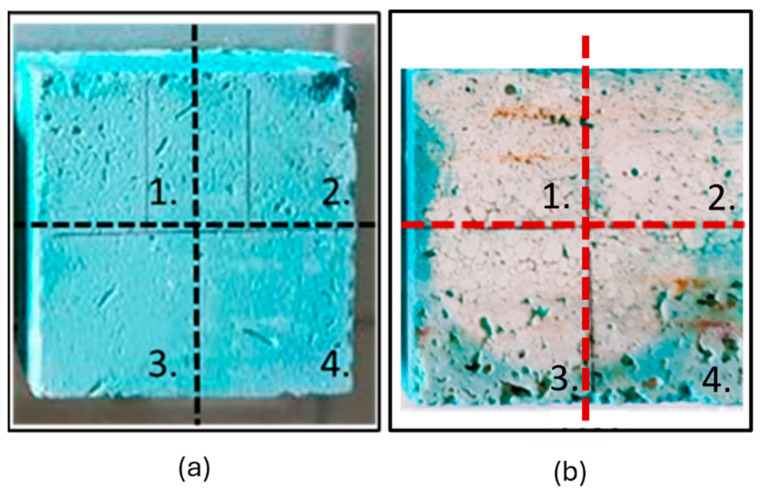
(**a**) Preliminary cleaning tests on the Travertine surface divided into four areas of applications: moving clockwise from 1 to 4, S2, S3, C2, and C3 formulations were applied, respectively; (**b**) Travertine surface after the application of the four formulations for 4 h.

Preliminary cleaning tests were carried out on three contaminated Travertine samples to estimate the best drying time to make the peeling process as easy as possible. Preliminary applications revealed the inadequacy of formulations S1 and C1 on the stone samples (rigid behaviour, the impossibility of peeling off, residuals, etc.), while S2, S3, C2, and C3 demonstrated superior performance from the outset. These findings were corroborated by rheological tests (see Section 2.1). Consequently, S1 and C1 were excluded from further experimentation (the test results are available in the Supplementary Materials, Figure S9), and all subsequent tests utilized only the formulations containing chelating agents (S2, S3, C2, and C3) which were divided into 4 areas (Figure 22). Three different drying times were tested: 2 h, 4 h, and 6 h. After a drying of 2 h, the gel-like layers were not dry enough to be removed by peeling, whereas after 6 h, they were excessively rigid and dry because of the excessive water evaporation. Therefore, the best drying time, which guaranteed a correct and easy peeling of the gel-like layer from the stone surface, turned to be 4 h. Among the four tested gel-like materials, C2 and C3 provided the best performance during peeling, and they left no macro-residues on the treated surfaces; hence, these two formulations were chosen for further cleaning tests on Travertine, Lecce stone, and Carrara Marble. An application grid made of 2 areas was built on each surface sample to test the cleaning action of the two formulations (C2, C3) with an optimized drying time of 4 h. 

### 4.5. NMR and SEM-EDS Monitoring of the Stones’ Contamination and Gel-like Formulations Cleaning

Portable NMR and SEM-EDS were used to monitor the contamination process and to assess the cleaning efficacy of the different gel-like formulations, loaded with the two chelators (EDTA and PST), in removing metal corrosion products from carbonate stone surfaces. Specifically, NMR measurements were performed, in a non-destructive and non-invasive way, using a portable spectrometer NMR-MOUSE equipped with an open magnet that generates a magnetic field of 0.35 T and a radiofrequency probe that receives the NMR signal coming from 2 mm deep below the sample surface. All samples of Travertine, Lecce stone, and Carrara marble, along with the portable NMR probe, were placed in an insulating box to keep the relative humidity (RH) and temperature (T) constant over time, avoiding oscillations [2,32]. Specifically, the RH and T correspond to those present in the room of the laboratory and were RH = (50.0 ± 3.5)% and T = (24.0 ± 1.0) °C, respectively. The microclimatic conditions in the insulating box were monitored with a TROTEC^®^ BC06 thermo-hygrometer. The spin-spin relaxation time *T*_2_, which is sensitive to water mobility and paramagnetic ions, was quantified with a Carr-Purcell Meiboom-Gill (CPMG) sequence [16,17] on the surface of the untreated and contaminated samples and of those cleaned with the gel-like materials. The CPMG sequence was characterized by a number of scans (NS) = 2048, repetition time (TR) = 0.5 s, number of echoes = 200, echo time (TE) = 0.03 s, and acquisition time (AT) = 25 min. TheMinispec^®^ software V2.59 Rev.26 was used to run and control the NMR experiments. 

A sampling procedure was required for SEM-EDS analyses. The samples were treated with graphite and analysed using an FEI Quanta 400 SEM equipped with an EDAX Genesis Microanalysis System. Prior to each treatment, the samples were examined in the presence of metal corrosion products and after cleaning. SEM-EDS also allowed us to characterize the chemical composition of the stones as well as that of the metallic corrosion products.

### 4.6. FTIR ATR Measurements of the Gel-like Layers

The Fourier Transform Infrared (FTIR) technique was used to obtain the spectroscopic characterization of the six different gel-like formulations (S1, S2, S3, C1, C2, C3). Then, FTIR analyses were also performed on the two gel-like formulations (C2 and C3) after being used to clean the stone surfaces to detect possible chemical interactions among atoms of the gel-like material and metal ions of the corrosion products. 

FTIR analyses were conducted with a JASCO FTIR410 spectrometer, utilizing the Attenuated Total Reflection (ATR) mode. The infrared beam emitted from a source undergoes numerous internal reflections within a non-absorbing ZnSe crystal. During each reflection, the quantum mechanical properties of the radiation generate an evanescent field at the crystal’s interface with the surrounding medium. This radiation serves to investigate the absorption spectrum of the sample, which arises from chemical bonds, particularly stretching and bending motions. The parameters used for recording each FTIR spectrum were as follows: 256 scans, employing a triangular apodization function, and a resolution of 2 cm^−1^.

## Figures and Tables

**Table 1:** Six different gel-like formulations synthesized using the same PVA–borax (4:1) mixture and different concentrations (% *w*/*w*) of EDTA, PST, AG.

Formulation Name	PVA (% *w*/*w*)	B-Water (% *w*/*w*)	EDTA (% *w*/*w*)	PST (% *w*/*w*)	AG (% *w*/*w*)
S1	4.0	1.0	-	-	-
S2	4.0	1.0	0.5	-	-
S3	4.0	1.0	-	1.0	-
C1	4.0	1.0	-	-	0.5
C2	4.0	1.0	1.5	-	0.5
C3	4.0	1.0	-	2.0	0.5

## Data Availability

The data presented in this study are available in this article.

## Supplementary Materials

The following supporting information can be downloaded at: https://www.mdpi.com/article/10.3390/gels10070455/s1, Figure S1: EDS spectra of Figure 5, from points 1 and 2 on stained Travertine with copper sulphate layer; Figure S2: EDS spectra of Figure 6, from points 1 and 2 on stained Lecce stone with copper sulphate layer; Figure S3: EDS of Figure 7 spectra from points 1 and 2 on stained Marble stone with copper sulphate layer; Figure S4: Treated Travertine: (a), (c) are respectively EDS spectra from points 1 and 2 after cleaning with C2 formulation; (b), (d) are respectively EDS spectra from points 1 and 2 after cleaning with C3 formulation. All EDS spectra are referred to Figure 11; Figure S5: Lecce Stone After Cleaning: (a), (c) are respectively EDS spectra from points 1 and 2 after cleaning with C2 formulation; (b), (d) are respectively EDS spectra from points 1 and 2 after cleaning with C3 formulation. All EDS spectra are referred to Figure 12; Figure S6: Carrara Marble After Cleaning: (a), (c) are respectively EDS spectra from points 1 and 2 after cleaning with C2 formulation; (b), (d) are respectively EDS spectra from points 1 and 2 after cleaning with C3 formulation. All EDS spectra are referred to Figure 13; Figure S7: FTIR spectra of S2 and S3 formulations before being used; Figure S8: FTIR spectra of S1 and C1 formulations before being used; Figure S9: (a) Area of the lithotype treated with metal corrosion patinas, treated with S1, (b) Area of the lithotype treated with metal corrosion patinas, treated with C1, (c) Application of S1, (d) Application of C1, (e) Removal of S1, (f) Removal of C1, (g) Area of the lithotype treated with metal corrosion patinas, after treatment with S1, (h) Area of the lithotype treated with metal corrosion patinas, after treatment with C1.
