# Supplementary material for: Multi-Technique Assessment of Chelators-Loaded PVA-Borax Gel-like Systems Performance in Cleaning of Stone Contaminated with Copper Corrosion Products
Source: Gels. 2024 Jul 11;10(7):455. doi: 10.3390/gels10070455 (PMC11276506; doi:10.3390/gels10070455)
Supplement: Supplementary file 1 [file gels-10-00455-s001.zip › gels-3056121-supplementary.pdf]

In this supplementary section, we present detailed EDS (Energy Dispersive X-ray Spectroscopy) spectra collected from various lithotypes before and after cleaning treatments. The samples were treated with graphite and analyzed using an FEI Quanta 400 SEM equipped with an EDAX Genesis Microanalysis System. Prior to each treatment, the samples were examined in the presence of metal corrosion products and again after cleaning. These spectra provide insight into the elemental composition of the surfaces, particularly focusing on the presence of copper sulphate and the effectiveness of cleaning formulations C2 and C3 in removing these deposits. The SEM-EDS analysis also characterized the chemical composition of the stones and the metallic patinas, highlighting the differences in elemental distribution and concentration. This offers a comprehensive view of the changes induced by the cleaning processes.

SEM-EDS monitoring of untreated and stained carbonate stones

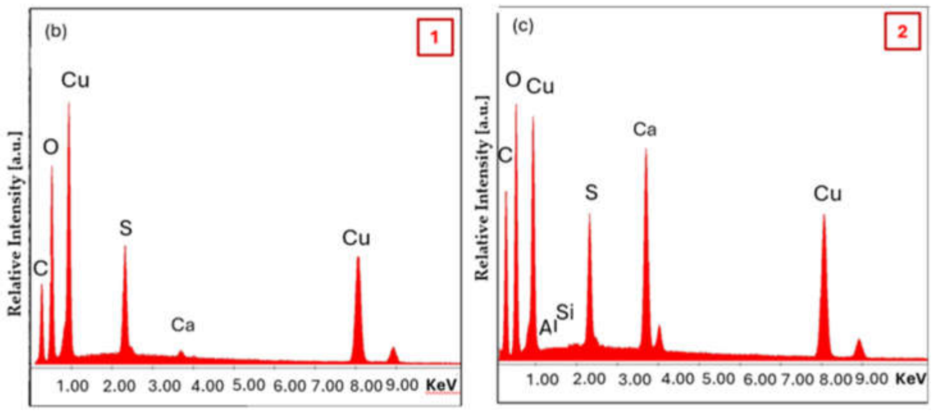

Figure 1 EDS spectra of Fig.5, from points 1 and 2 on stained Travertine with copper sulphate layer.

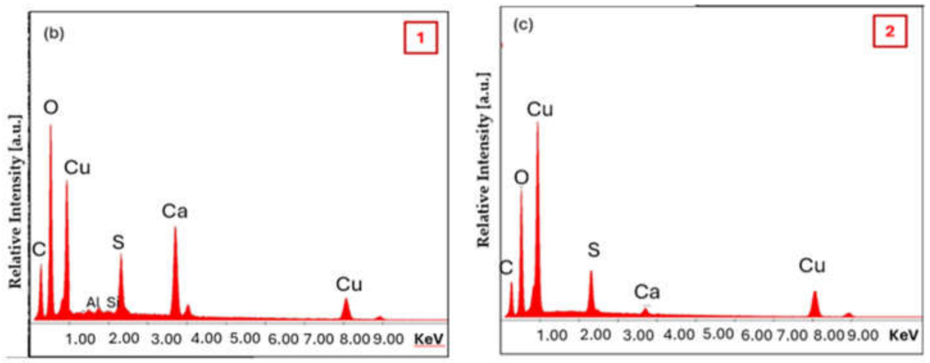

Figure 2 EDS spectra of Fig.6, from points 1 and 2 on stained Lecce stone with copper sulphate layer.

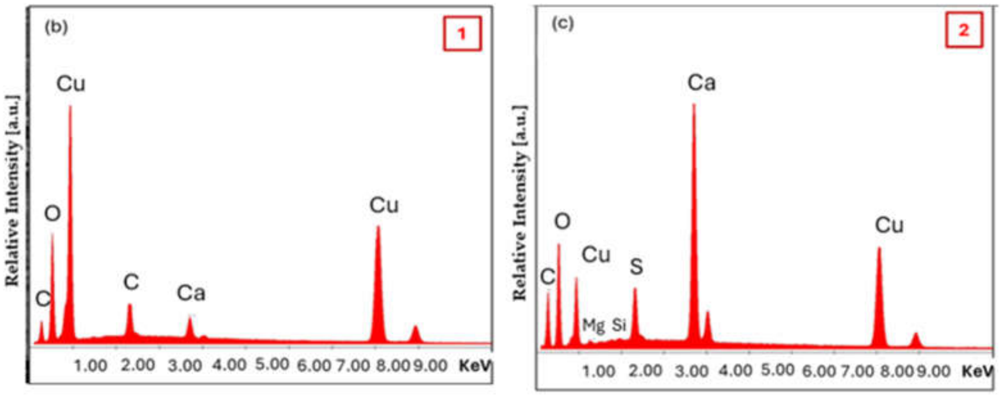

Figure 3 EDS of Fig. 7 spectra from points 1 and 2 on stained Marble stone with copper sulphate layer.

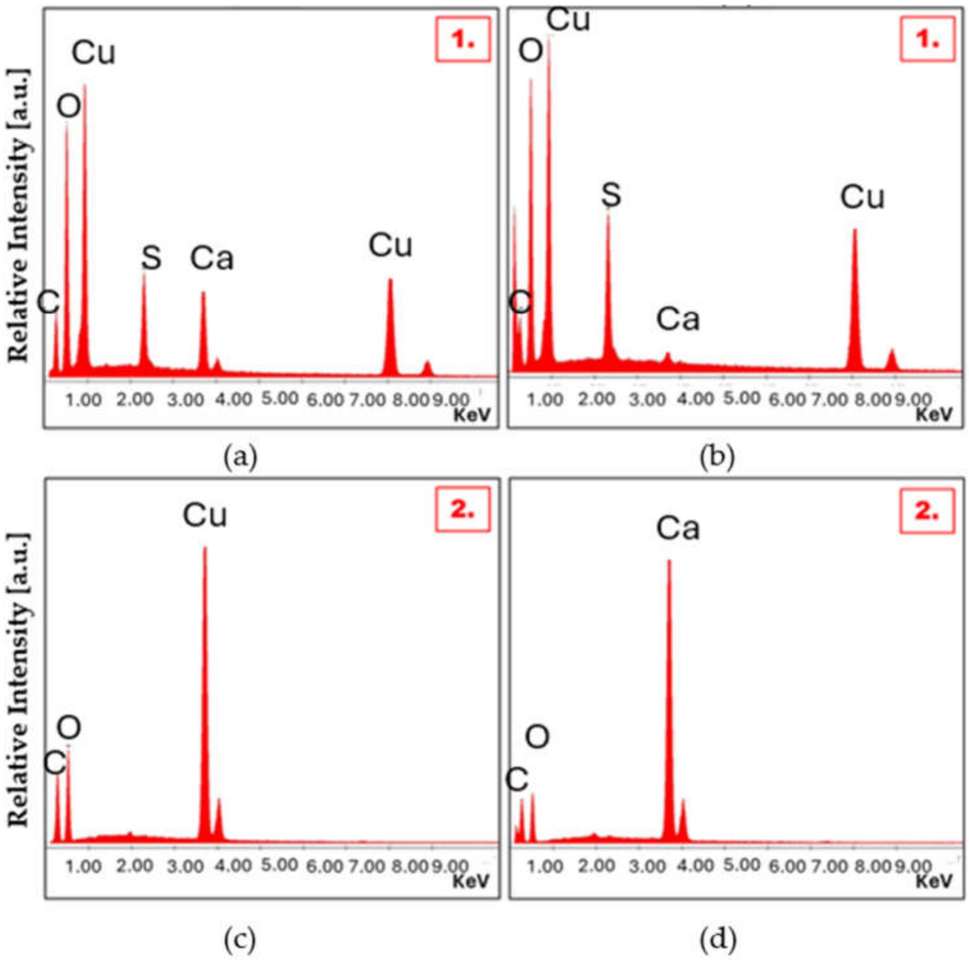

Figure 4 Treated Travertine: (a), (c) are respectively EDS spectra from points 1 and 2 after cleaning with C2 formulation; (b),(d) are respectively EDS spectra from points 1 and 2 after cleaning with C3 formulation. All EDS spectra are referred to Fig. 11.

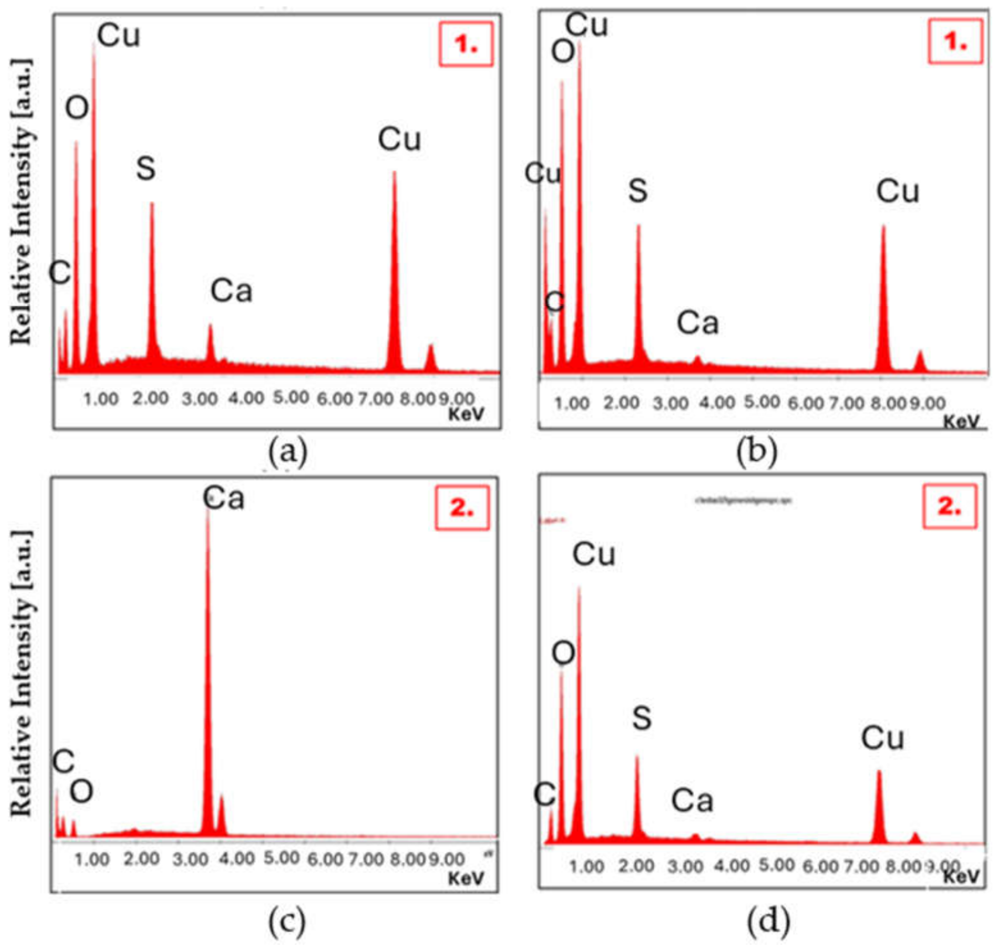

Figure 5. Lecce Stone After Cleaning: (a), (c) are respectively EDS spectra from points 1 and 2 after cleaning with C2 formulation; (b),(d) are respectively EDS spectra from points 1 and 2 after cleaning with C3 formulation. All EDS spectra are referred to Fig. 12.

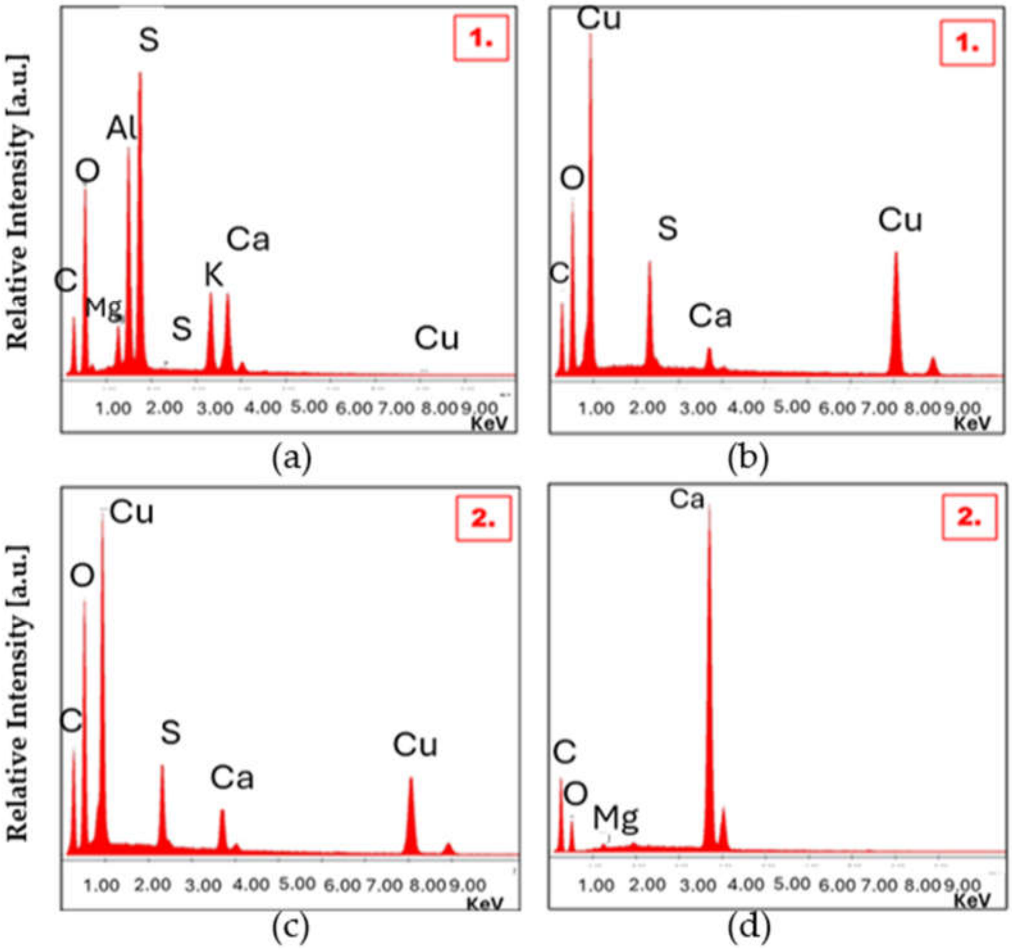

Figure 6. Carrara Marble After Cleaning: : (a), (c) are respectively EDS spectra from points 1 and 2 after cleaning with C2 formulation; (b),(d) are respectively EDS spectra from points 1 and 2 after cleaning with C3 formulation. All EDS spectra are referred to Fig. 13.

The following figures depict EDS spectra of various lithotypes before and after treatment with formulations C2 and C3. The analysis reveals copper sulphate crystals and demonstrates the efficacy of the cleaning gels in their removal. These spectra offer valuable insights into the elemental composition of the stones and the metallic patinas both pre- and post-treatment

FTIR study of S2, S3, S1, C1 formulations before being used

The supplementary materials present an FTIR study focused on formulations S2 and S3, providing detailed insights into their chemical compositions and structural characteristics. This analysis offers a comprehensive understanding of the formulations' molecular properties and their potential applications.

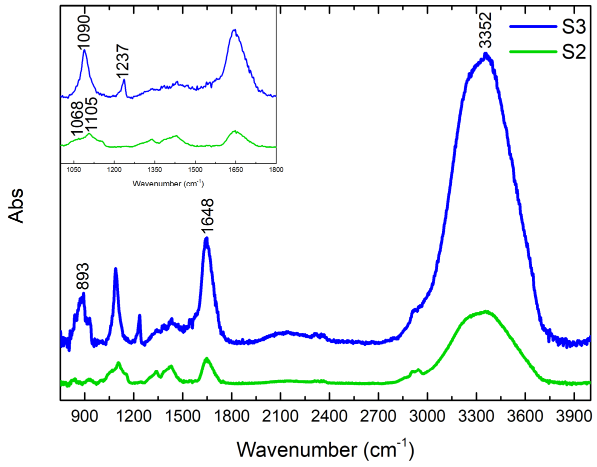

Figure 7 FTIR spectra of S2 and S3 formulations before being used.

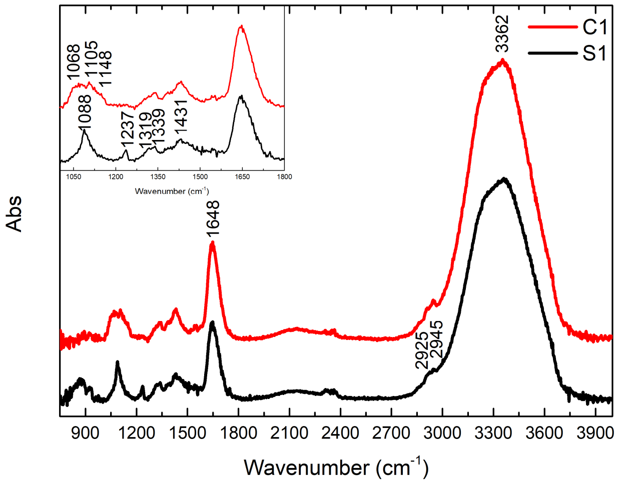

Figure 8. FTIR spectra of S1 and C1 formulations before being used.

Cleaning tests of carbonates samples

We conducted preliminary cleaning tests using all formulations. Similar to S2 and S3, which are High Viscous Polymeric Dispersions, formulations S1 and C1 proved to be unsuitable for removal by peeling from porous stone surfaces. This is because S1 and C1 do not come off easily and uniformly; they break during peeling and leave macroscopic residues.

When applied to porous stone surfaces, S1 did not peel off smoothly. Instead, it broke into pieces

during the peeling process, leaving behind macroscopic residues. This residue compromises the effectiveness of the cleaning process.
Similar to S1, C1 also faced issues with peeling. It did not come off uniformly and broke apart, leaving significant macroscopic residues on the stone surface. This made it unsuitable for effective cleaning.

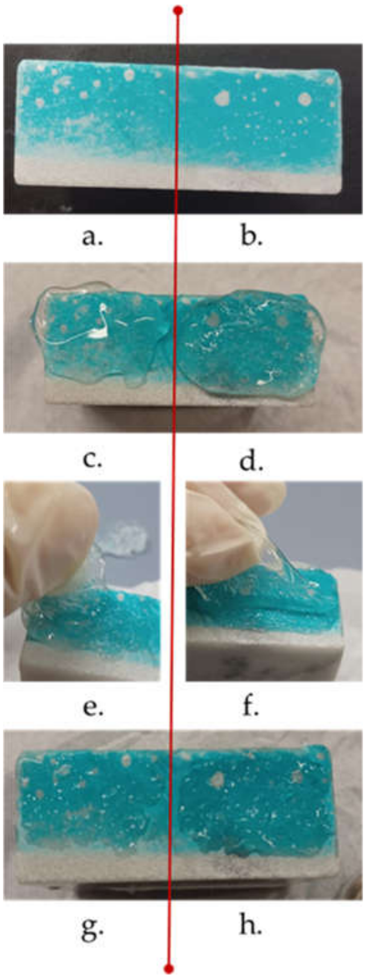

Figure 9. a. Area of the lithotype treated with metal corrosion patinas, treated with S1 b. Area of the lithotype treated with metal corrosion patinas, treated with C1 c. Application of S1 d. Application of C1 e. Removal of S1 f. Removal of C1 g. Area of the lithotype treated with metal corrosion patinas, after treatment with S1 h. Area of the lithotype treated with metal corrosion patinas, after treatment with C1.

These preliminary tests highlight the limitations of formulations S1 and C1 for cleaning porous stone surfaces due to their poor peeling performance and residue issues. In contrast, while formulations S2 and S3 fared better, they also faced challenges. Therefore, further research is needed to develop more effective cleaning formulations for porous materials.
